# A novel bacterial symbiont in the seaweed *Gracilaria tenuistipitata* and its promising anti-inflammatory and antiproliferative potential in relation to special secreted metabolites

**DOI:** 10.3389/fmicb.2026.1935663

**Published:** 2026-09-29

**Authors:** Anh Hien Le, Le Na Thi Nguyen, Thanh Tien Tran, Lien Thuy Nguyen, Duc Anh Nguyen, Kien Kieu Trung, Phuong Ha Vu, Thao Kim Nu Nguyen, Thanh Lai Nguyen, Ha Do Minh, Tung Thanh Nguyen, Huy Huu Quoc Ho, Luyen Thuy Thi Bui, Toan Linh Nguyen, Hai The Pham

**Affiliations:** 1 Research group for Physiology and Applications of Microorganisms (PHAM group), GREENLAB, Center for Life Science Research (CELIFE), Faculty of Biology, VNU University of Science - Vietnam National University, Hanoi, Vietnam; 2 Faculty of Biology, VNU University of Science - Vietnam National University, Hanoi, Vietnam; 3 Faculty of Pharmacognosy and Traditional Medicine, Hanoi University of Pharmacy, Hanoi, Vietnam; 4 Faculty of Pharmaceutical Chemistry and Technology, Hanoi University of Pharmacy, Hanoi, Vietnam; 5 Department of Pathophysiology, Vietnam Military Medical University, Hanoi, Vietnam; 6 College of Health Sciences, VinUniversity, Hanoi, Vietnam; 7 Center for Innovations in Health Sciences, VinUniversity, Hanoi, Vietnam; 8 Vietnamese German Center for Medical Research (VG-CARE), Hanoi, Vietnam

**Keywords:** anti-inflammatory, antiproliferative, *Bacillus*, *Gracilaria tenuistipitata*, LC-HRMS, surfactin, zebrafish toxicity

## Abstract

Marine macroalgae host compositionally complex microbiomes that constitute an evolutionarily refined, yet largely unexplored source of structurally diverse natural products. Thus, the bioactivities of the algae may be related to those of their associated microorganisms. Here we describe the discovery of strain S.100, an endophytic bacterium isolated from the red alga *Gracilaria tenuistipitata* and displaying remarkable anti-inflammatory and antiproliferative activities, through a primary screening on ethyl acetate (EtAc), chloroform and n-hexane extracts of its culture. S.100 was taxonomically identified to the species level by morphology observation, and sequence analyses of 16S rRNA and housekeeping rpoB genes. Its anti-inflammatory and antiproliferative activities, as well as toxicity, were further investigated by using standard procedures performed on the EtAc extract. This extract was also fractionated to five fractions and the fraction with the most pronounced activities was subject to liquid chromatography–high-resolution mass spectrometry (LC-HRMS) to identify its chemical components. The results show that S.100 belongs to a *Bacillus* species reported for the first time to be associated with *Gracilaria tenuistipitata*. The EtAc extract of its culture selectively inhibited proliferation of human cervical (HeLa, IC_50_ = 31.87 μg/mL) and hepatocellular (HepG2, IC_50_ = 63.8 μg/mL) carcinoma cell lines, with substantially lower cytotoxicity toward normal human embryonic kidney cells (HEK 293T, IC_50_ = 181.1 μg/mL). At 120 μg/mL, the extract also suppressed lipopolysaccharide-induced nitric oxide production in RAW264.7 macrophages. Toxicological assessment in zebrafish embryos revealed no developmental abnormalities below 200 mg/L and an LC50 of 421.6 mg/L at 96 h, consistent with a favorable safety profile. Bioactivity-guided fractionation, coupled with LC-HRMS analysis, putatively annotated 16 specialized metabolites in the most active fraction (fraction 4), including noteworthily bioactive cyclic lipopeptides (surfactins B and C), β-carboline and quinoline alkaloids (norharmane, harmane, and quinoline-4-carboxylic acid), and phenolic acids (gallic and 2,3-dihydroxybenzoic acids). Together, these findings show exciting biopharmaceutical application potential of S.100 and highlight macroalgal endophytes as a promising chemical source for the discovery of marine-derived therapeutics.

## 1 Introduction

*Gracilaria tenuistipitata* is a significant species within the genus *Gracilaria*, which is part of the Gracilariaceae family of red algae. The genus *Gracilaria* comprises a prominent group of marine algae with notable economic and biological value; its species diversity in the Asian region was extensively classified and documented by Chang and Xia (1976). This algal species is of considerable economic importance, particularly in the production of agar, and is recognized as a critical resource in various Asian countries. *G. tenuistipitata* exhibits a broad range of applications in the biopharmaceutical field, as this species demonstrates various pharmacological activities, including antibacterial, antifungal, antiviral, anti-inflammatory, antioxidant, anticancer, anti-hyperlipidemic, and anticholinesterase activities (Vivek et al., 2022; Yang et al., 2012; Yeh et al., 2012). Furthermore, it has been shown to exert beneficial effects on the immune system and metabolism in cells (Chen et al., 2013), which may enhance overall health and support the body’s ability to fight infections and diseases. During its life cycle, a seaweed such as *G. tenuistipitata* accumulates considerably rich and diverse bioactive compounds in its biomass (Aziz et al., 2021). This phenomenon is hypothetically partially due to the presence of a highly diverse symbiotic microbiome that has co-evolved with the seaweed over millions of years.

Previous research on the microbiomes of algae indicates that they comprise bacteria commonly belonging to the phyla *Proteobacteria, Bacteroidetes, Actinobacteria*, and *Cyanobacteria* (Gu et al., 2023; Mancuso et al., 2016). Macroalgae-associated microorganisms play essential roles in algal metabolism, protection against pathogenic bacteria, nutrient assimilation, and the regulation of algal growth (Zhang et al., 2025). These microorganisms produce secondary compounds that not only support the growth, development, and adaptation of algae but also have important biopharmaceutical activities (Variem and Kizhakkedath, 2021). Moreover, these microorganisms can stimulate algae to produce secondary compounds with biopharmaceutical activities (Khandwal et al., 2025; Manam et al., 2025; Variem and Kizhakkedath, 2021). The symbiosis between algae and bacteria evolved during life and in the phycosphere environment can increase the production of beneficial compounds for both bacteria and algae, and create bioactive compounds with pharmaceutical application potential (Variem and Kizhakkedath, 2021). In addition, previous studies have shown that bacteria symbiotic with algae can synthesize enzymes and byproducts with antibiotic activities, helping to protect algae from pathogens in the marine environment (Chukwudulue et al., 2023). Furthermore, they may influence the synthesis and distribution of complex polysaccharides and carotenoids in seaweed cells, contributing to anti-inflammatory, antioxidant and immunomodulatory activities of seaweed hosts (Matin et al., 2024; Menaa et al., 2020).

In red algae, such as *G. tenuistipitata*, the associated microbial community can be very diverse, and include bacteria belonging to various phyla such *as Proteobacteria, Bacteroidetes, Actinobacteria*, and *Firmicutes*. These bacteria often participate in the biological interactions, polysaccharide breakdown and metabolism of algae (Gu et al., 2023). However, to date, research data on the microbial communities associated with red algae has been still very limited, despite numerous studies on characterization and bioactive compounds of red algae species themselves. A similar situation happens with *G. tenuistipitata*, as no studies have been yet reported on microorganisms associated with this algal species, especially in terms of their correlations in bioactivities and producing bioactive compounds.

In this study, through a primary screening, we discovered a symbiotic bacterial strain with remarkable anti-inflammatory and antiproliferative activities from the red alga *G. tenuistipitata*. We also taxonomically identified the strain to the species level and found that it belongs to a *Bacillus* species that is reported for the first time to be associated with *G. tenuistipitata*. An in-depth study of anti-inflammatory and antiproliferative activities of the strain with a preliminary identification of the associated metabolites it produces were also carried out. Thus, we provide the first published scientific data on a microorganism associated with *G. tenuistipitata* and its biological activities. The results also imply the potential applications of the strain and its bioactive compounds in biopharmaceutical context.

## 2 Materials and methods

### 2.1 Materials and microbial strains

The chemicals used in this study were purchased from credited suppliers as follows: Dulbecco’s Modified Eagle Medium (DMEM) Low Glucose (Gibco, USA), Fetal Bovine Serum (FBS) (Gibco, USA), Penicillin Streptomycin (PS) (Gibco, USA), Phosphate Buffered Saline (PBS) (Gibco, USA), TrypLE (Gibco, USA), MTT Assay Kit (Gibco, USA), Dimethyl sulfoxide (DMSO) (Fisher, USA), 96% Ethanol (Merck, USA), Lipopolysaccharide (LPS) (Santa Cruz Biotechnology, USA), and the NO Griess Reagent Kit from Invitrogen (USA), Sodium Ethylate (EtNa) (Vietnam), DNA suspension (Vietnam), 2 × Taq Master Mix (Vazyme, China), EcoDye Nucleic Acid Staining Solution (Suran, Vietnam), and Gene Ruler 1kb DNA Ladder (Thermo, USA).

Cell lines used were RAW264.7 (murine macrophage TIB-71), HEK 293T (CRL-3216™), HepG2 (HB-80065™), and HeLa (CCL-2).

The algae-associated bacterial strains used for screening in this study (Table 1) were 13 strains that were suspected to be endophytic *Bacillus* bacteria previously isolated by our group from different macroalgae collected from Cat Ba Island (Hai Phong, Vietnam) (see Supplementary material for details about our isolation method of the endophytes). (We aimed at *Bacillus* bacteria because this group is known to be a rich source of bioactive compounds).

**TABLE 1:** The collection of algae-associated bacterial strains used for screening in this study.

No.	Strains	Algal source
1	S.002	*Gelidium crinale*
2	S.051	*Ulva flexuosa*
3	S.100	*Gracilaria tenuistipitata*
4	S.234	*Dermonema pulvinatum*
5	S.288	*Gracilaria tenuistipitata*
6	S.354	*Centroceras clavulatum*
7	S.374	*Valonia fastigiata*
8	S.408	*Chaetomorpha linum*
9	S.435	*Chaetomorpha linum*
10	S.467	*Cladophora aokii*
11	S.556	*Gracilaria tenuistipitata*
12	S.579	*Dermonema pulvinatum*
13	S.593	*Dermonema pulvinatum*

### 2.2 Morphological characterization of the strain of interest

Colony morphology of the strain was assessed using the T-streak method on Luria-Bertani (LB) agar plates. The bacterial cell morphology was determined by Gram staining, following the protocol outlined by Hucker and Conn (1923), with *Escherichia coli* and *Bacillus subtilis* strains serving as controls. The stained cells were examined under an optical microscope (Nikon Eclipse Ei R, Japan) with an oil immersion objective at 100 × magnification, and images were captured for subsequent analysis.

### 2.3 Bacterial identification using 16S rRNA gene analysis and housekeeping genes

Macroalgae-associated microbiomes are complex and dynamic communities living either on the algal surface (as epiphytes) or within the surrounding phycosphere. The extraction of colony DNA of the strain of interest was done using the EtNa method employed by Vingataramin and Frost (2015). Polymerase chain reaction (PCR) was performed using the Nexus GSX1 PCR instrument (Eppendorf, Germany). The primer sequences for the 16S rRNA gene were: 5’-AGAGTTTGATCCTGGCTCAG-3’ (forward) and 5’-GGTTACCTTGTTACGACTT-3’ (reverse). The thermal cycling conditions consisted of an initial denaturation at 94 °C for 2 min, followed by 35 cycles of denaturation at 94 °C for 45 s, annealing at 55 °C for 45 s, and extension at 68 °C for 2 min, with a final extension step at 68 °C for 7 min.

For the identification of *Bacillus* species, a partial *rpoB* gene was amplified by PCR, as described by Ki et al. (2009), using the forward primer rpoB1206 (5’-ATCGAAACGCCTGAAGGTCCAAACAT-3’) and the reverse primer rpoB3202 (5’-ACACCCTTGTTACCGTGACGACC-3’). The thermal cycling protocol consisted of an initial denaturation at 95 °C for 3 min, followed by 35 cycles of denaturation at 95 °C for 20 s, annealing at 55 °C for 30 s, and extension at 72 °C for 90 s, with a final extension at 72 °C for 5 min. The PCR products were sent for sequencing at Genlab (Vietnam). Sequence analysis was carried out using NCBI BLAST, and a phylogenetic tree was constructed using MEGA X software.

### 2.4 Preparation of bacterial medium extracts

Strain S.100 was cultured in Yeast Extract-Soluble Starch medium (YS) at 30 °C for 72 h. The metabolites from the culture broth were extracted using solvents of increasing polarity: n-hexane, chloroform, and ethyl acetate (EtAc) (Mohan et al., 2016). For each solvent, the extraction was carried out at a 1:1 (v/v) ratio. The mixture was then centrifuged at 4,000 rpm for 15 min to facilitate phase separation, and the organic phase was collected. This extraction process was repeated three times to ensure maximum recovery of soluble compounds. The pooled solvent extracts were evaporated to dryness using a rotary vacuum evaporator (model RE300, Stuart, United Kingdom). The resulting crude extracts were stored at −20 °C and dissolved in 100% DMSO prior to subsequent experiments.

### 2.5 Cell viability assay

Human cervical cancer cells (HeLa) and human liver cancer cells (HepG2) were cultured in 96-well plates at a density of 5.0 × 10^3^ cells per well in low-glucose DMEM medium, while human embryonic kidney cells (HEK 293T) were cultured in high-glucose DMEM medium supplemented with 1% penicillin-streptomycin and 10% fetal bovine serum at 37 °C and 5% CO_2_. Following a 24-h period, the cells were exposed to treatment with the extract of interest (dissolved in DMSO as described above) at a range of concentrations and were cultured continuously for a total of 48 h. Concentration ranges were adjusted per cell line based on preliminary sensitivity testing (see Supplementary Tables 1–6). The negative control samples were DMSO 0.1% and medium. Finally, 10 μL of a dye solution containing the MTT labeling reagent, calibrated to a final concentration of 0.25 mg/mL, was added to each well. After a subsequent incubation and solubilization, the absorbance of the cells was measured at 570 nm using an ELISA plate reader (BioTech Power Wave XS, Winooski, VT, USA) to estimate the cell viability rate of the extract in comparison with control samples. The positive control was doxorubicin. The experiments were performed with technical triplicates within each of three independent biological experiments. Data are presented as mean ± SEM of three independent biological experiments (*n* = 3). Cell viability data of each biological repeat would be normalized to the vehicle control data. IC_50_ was calculated using built-in function of Graphpad Prism 10.5.0, in particular we used four-parameter logistic model with a constrained bottom of 0. The CI95 were also determined by the same method and are reported in Table 2.


$$Cell\;Viability\left(\%\right)=\left(\frac{O\,D\,\_\,s\,a\,m\,p\,l\,e}{O\,D\,\_\,c\,o\,n\,t\,r\,o\,l}\right)x100$$

**TABLE 2:** Preliminary screening for antiproliferative and anti-inflammatory activities.

No.	Name	Antiproliferative activities		Anti-inflammatory activities
		Against HeLa	Against HepG2	
1	S.002	IC_50_ = 76.26 (95% CI: 64.23 to 91.40)	IC_50_ = 115 (95% CI: 98.54 to 136.2)	−
2	S.051	−	−	−
3	S.100	IC_50_ = 31.87 (95% CI: 24.60 to 42.18)	IC_50_ = 63.8 (95% CI: 55.20 to 72.70)	+
4	S.234	−	−	−
5	S.288	−	−	−
6	S.354	−	−	−
7	S.374	−	−	−
8	S.408	−	−	−
9	S.435	−	−	−
10	S.467	−	−	+
11	S.556	−	−	−
12	S.579	−	−	−
13	S.593	−	−	−

“−” indicates weak activity, “+” indicates strong activity (*P* < 0.05).

The Selectivity Index (SI) was calculated as SI = IC_50_ (non-cancerous line)/IC_50_ (cancer line) (Mahavorasirikul et al., 2010).

### 2.6 NO assay

The anti-inflammatory activity of a strain of interest was assessed by the NO assay, which measures nitric oxide generation in the RAW264.7 macrophage cell line, according to previously published procedures with minor changes (Potter et al., 2005). Briefly, in 96-well plates, RAW264.7 cells were seeded at a density of 30,000 cells per well in high-glucose DMEM, supplemented with 10% FBS and 1% penicillin-streptomycin, incubated at 37 °C, and supplied with 5% CO_2_. Then, 90 μL of the extract of interest (dissolved in DMSO as described above) was added into each well to achieve a final concentration of 120 μg/mL. To induce inflammation, 20 μL of LPS (2 μg/mL) was added after 2 h. Following the manufacturer’s directions, the NO Griess Reagent Kit was used to assess the anti-inflammatory effects after 48 h. The experiments were performed with technical triplicates within each of three independent biological experiments. Data are presented as mean ± SEM of three independent biological experiments (*n* = 3).

### 2.7 Zebrafish husbandry and zebrafish toxicological tests

A recirculation system from Thoren (Thoren Aquatic Systems, USA) was used for housing the adult Danio rerio (AB strain), which commonly serves as a standard model in toxicological research (Jayasinghe and Jayawardena, 2019). The system was maintained at a temperature of 27.0 °C ± 1 °C, with a light/dark photoperiod of 14 h of light and 10 h of dark. The fish were fed twice daily with commercial food, including Tetramin Flakes (Tetra, Spectrum Brands, USA) or MEM prime (BernAqua, Belgium).

Two males and three females were placed in breeding tanks to obtain fertilized eggs. Every morning, the eggs were collected and gently rinsed to remove any debris. Embryos of zebrafish were observed by using microscopy (model MZ7.5, Leica, Germany), and until the embryo divided into 4–8 cells, it was transferred to E3 medium (containing 5 mM NaCl, 0.17 mM KCl, 0.33 mM CaCl_2_, and 0.33 mM MgSO_4_) and selected for subsequent testing.

Following the OECD testing guideline 236 and our previous research (Nguyen et al., 2024), the extract of interest (dissolved in DMSO as described above) was evaluated for cytotoxicity on zebrafish embryos. In essence, 20 embryos were treated with 5 mL of test solution on 6-well polypropylene plates (SPL Sciences). Six concentrations of the extract were tested, including: 100, 200, 400, 500, 1,000 and 2000 mg/L). A daily stereomicroscope (model MZ7.5, Leica, Germany) would be employed to assess mortality, hatching status, and any developmental malformations. Dead embryos and larvae were removed from plates to maintain water quality. At least three replications of the toxicological assay were conducted, and each test was stopped after 96 h.

### 2.8 Fractionation of the extract of interest

The extract was thoroughly mixed with silica gel at a 1:2 (w/w) ratio, and the mixture was evaporated to dryness using a rotary evaporator (model RE300, Stuart, United Kingdom) at 55 °C for 30 min. This dried sample–silica mixture was then dry-loaded onto a chromatography column prepacked with a silica gel slurry (prepared at a ratio of 1 g silica to 2 mL solvent). The column was equilibrated for 2–3 h prior to elution. Step-gradient elution was performed sequentially using n-hexane:acetone mixtures (each containing 200 mL of each component solvent) at ratios of 100:1, 50:1, 30:1, and 10:1 (v/v), followed by dichloromethane:methanol mixtures at 30:1, 10:1, 5:1, and 2:1 (v/v). The flow rate was maintained at 0.5 mL/min throughout the separation process. Fractions were monitored by thin-layer chromatography (TLC), and those exhibiting similar Rf values were combined. The collected fractions were subsequently concentrated using the rotary evaporator.

### 2.9 LC-HRMS analysis

Liquid chromatography–high-resolution mass spectrometry (LC-HRMS) was performed using an Ultimate 3000 Ultra High-Performance Liquid Chromatography (UPLC) system coupled with an Exactive High-Resolution Mass Spectrometry (HRMS) detector. The fraction of interest was dissolved in methanol to a concentration of 0.1 mg/mL for liquid chromatography-mass spectrometry analysis. The analytical column ACQUITY UPLC BEH C18 (1.7 μm; 2.1 mm × 150 mm) (Waters, USA) was used for LC-HRMS analysis. For the mobile phase, two solvents were used, including Solvent A (0.1% formic acid in water) and Solvent B (0.1% formic acid in acetonitrile). The gradient elution process includes the following steps: 0–1.0 min, 95.0% A; 1.0–16.0 min, 95.0%–0.5% A; 16.0–26.0 min, 0.5% A; 26.0–26.2 min, 0.5%–95.0% A; 26.2–30.0, 95% A. The flow rate inside the column was set at 0.2 mL/min at a temperature of 40 °C. A preprogrammed syringe volume of 10 μL was set in an automated sample injection system. MS1 and MS2 data were analyzed over an m/z in the range about 100–1,000. The five most intense non-target precursor ions per scan cycle were chosen for fragmentation in order to acquire MS2 spectra utilizing an automatic data-dependent acquisition (DDA) mode. A 40% normalized collision energy (NCE) was used. Positive and negative electrospray ionization (ESI) modes were used to collect data in separate runs. Data-dependent MS^2^ (dd-MS^2^) scans were obtained at a resolution of 17,500 FWHM at m/z 200, whereas complete MS scans were obtained at a resolution of 70,000 FWHM at m/z 200. The sample was analyzed in duplicate.

Raw mass spectrometry data were recorded, processed and analyzed to identify the chemicals in the fraction of interest, following standard procedures suggested by the manufacturer of the equipment (details in Supplementary material).

### 2.10 Statistical analysis

The results are expressed as the mean ± standard error of the mean (SEM). Each experiment was performed in triplicate, and the data were statistically analyzed using appropriate methods. Statistical significance was assessed using analysis of variance (ANOVA) and Student’s *t*-test, as appropriate, with *p* < 0.05 considered statistically significant.

## 3 Results

### 3.1 Preliminary screening

The 13 algae-associated bacterial strains in our collection were evaluated for their anti-cancer and anti-inflammatory activities, through testing the ethyl acetate, chloroform and n-hexane extracts of their cultures. Noticeably, except for the EtAc extracts, the other extracts showed almost no activity (data not shown). The results with the EtAc extracts (Table 2) showed that among the 13 strains, only two strains (S.002 and S.100) displayed antiproliferative activities and two strains (S.100 and S.467) displayed anti-inflammatory activities. Thus, only strain S.100 was selected for further study because it well-displayed both activities with the lower IC_50_ values, indicating stronger cytotoxic potency, and hence a promising pharmaceutical value.

### 3.2 Identification of strain S.100

The morphological characteristics of strain S.100 indicate that it is a Gram-positive bacterium, with rod-shaped cells and round, opaque colonies when grown on LB agar plates (Figure 1).

**FIGURE 1:**
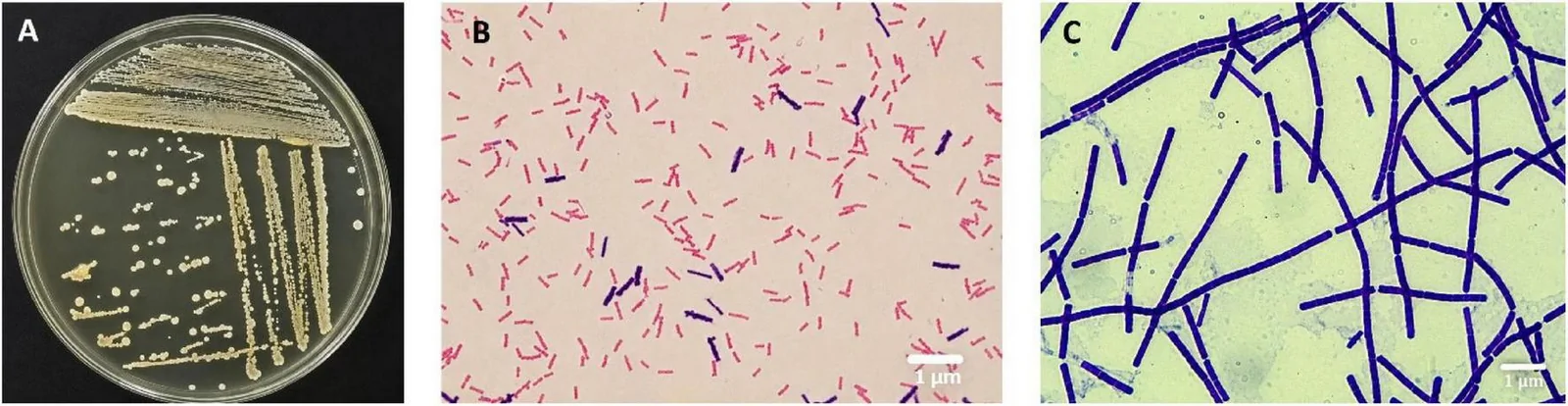
Morphology of strain S.100. **(A)** Colony morphology, **(B,C)** Gram staining results with *Bacillus subtilis* and *Escherichia coli*, respectively.

Using the NCBI GenBank database, the 16S rRNA gene sequence of S.100 was found to be 100% similar to those of the members of the genus *Bacillus*, and to three *Bacillus stercoris* strains in particular (Figure 2). Consequently, based on cell morphology, colony characteristics, and its 16S rRNA gene sequence, strain S.100 was identified as a *Bacillus* species.

**FIGURE 2:**
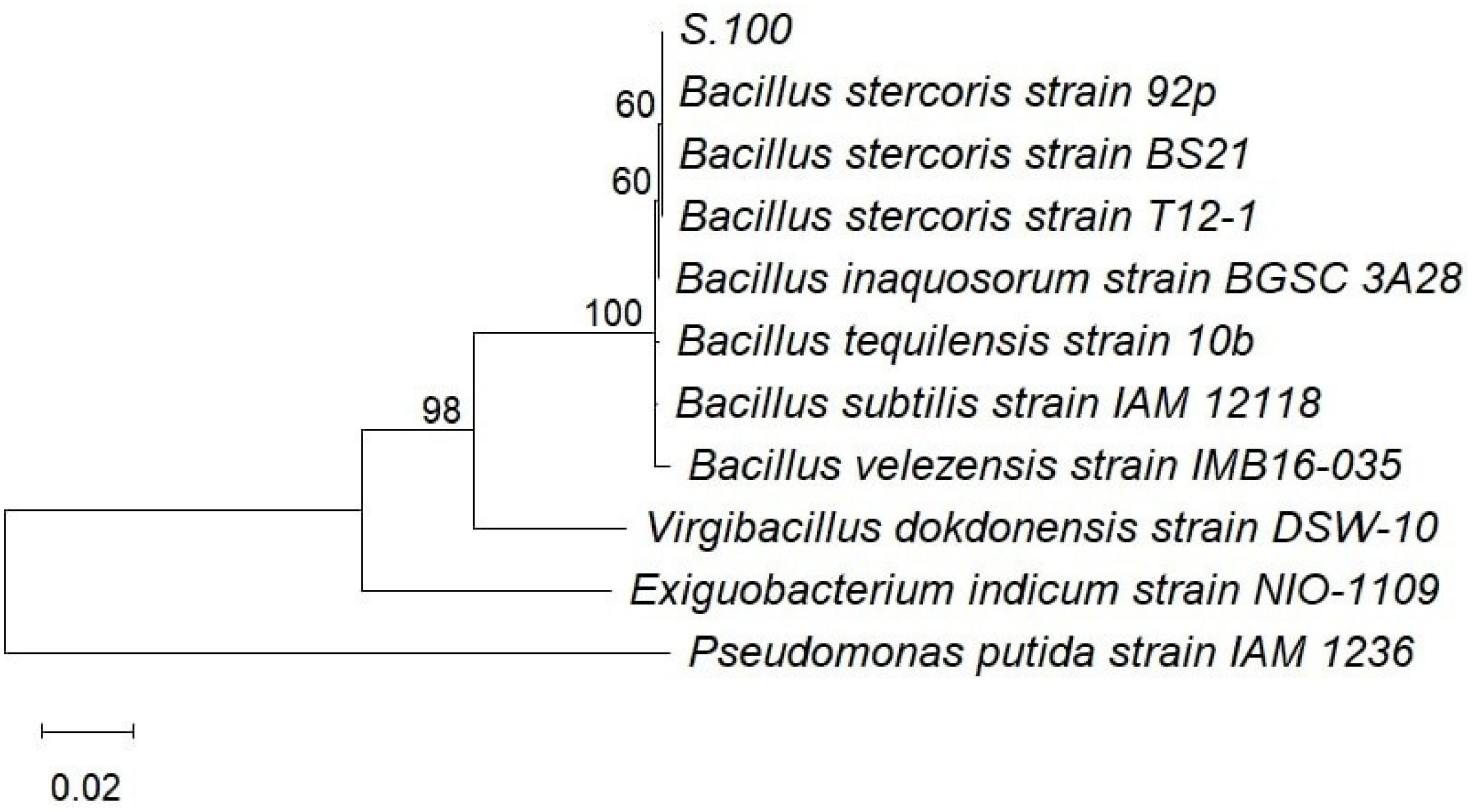
Phylogenetic tree of S.100 constructed based on the sequence alignment of the 16S rRNA gene. The numbers on the nodes indicate bootstraps values (of 1,000 replicates).

For further identification of strain S.100, the housekeeping gene (rpoB) was analyzed. The rpoB gene is one of the commonly used housekeeping genes for distinguishing species within the Bacillus genus, with a threshold value of approximately 98% (Adékambi et al., 2008). The analysis result showed that the rpoB sequence of strain S.100 was over 98% similar to *B. stercoris* (Figure 3 and Table 3). This result well aligns with that of the 16S rRNA gene analysis. Consequently, strain S.100 has been firmly identified as *B. stercoris*.

**FIGURE 3:**
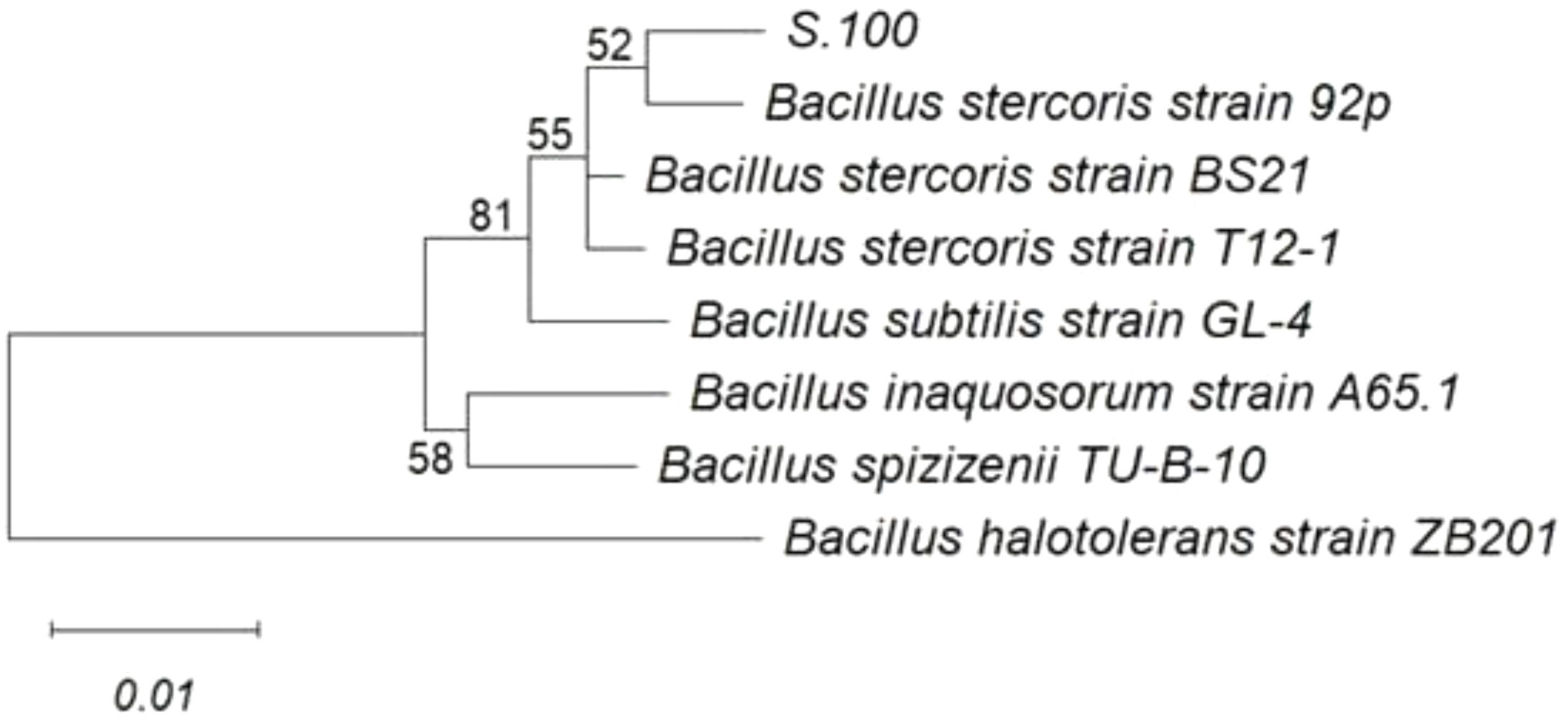
Phylogenetic tree of strain S.100 constructed based on the sequence alignment of the housekeeping gene *rpoB.* The numbers on the nodes indicate bootstraps values (of 1,000 replicates).

**TABLE 3:** Homology of the *rpoB* gene sequence of strain S.100 with those of several *Bacillus stercoris* strains from the NCBI database.

Strains compared	Sequence similarity (%)
*Bacillus stercoris* strain 92p	99.15
*Bacillus stercoris* strain BS21	98.96
*Bacillus stercoris* strain T12-1	98.86

### 3.3 Bioactivities of compounds secreted by strain S.100

To evaluate bioactivities of the metabolites secreted by S.100, we used the EtAc extract of its culture broth (S.100 EtAc in brief) as this was the only extract displaying the most activities during the preliminary screening (Supplementary Figure 1).

#### 3.3.1 Cytotoxicity of S.100

As shown in Figure 4, S.100 EtAc inhibited cancer cell growth in a concentration-dependent manner. S.100 EtAc markedly induced a higher cytotoxicity to both cancer cell lines (HeLa and HepG2) than the normal cells such as HEK 293T. The IC_50_ values of S.100 EtAc for HeLa (31.87 μg/mL) and HepG2 (63.8 μg/mL) were 5.7 and 2.8-fold lower, respectively, than that for HEK 293T (181.1 μg/mL) (95% CI presented in Table 2). [The result with a positive control sample (Doxorubicin) is shown in Supplementary Figure 2].

**FIGURE 4:**
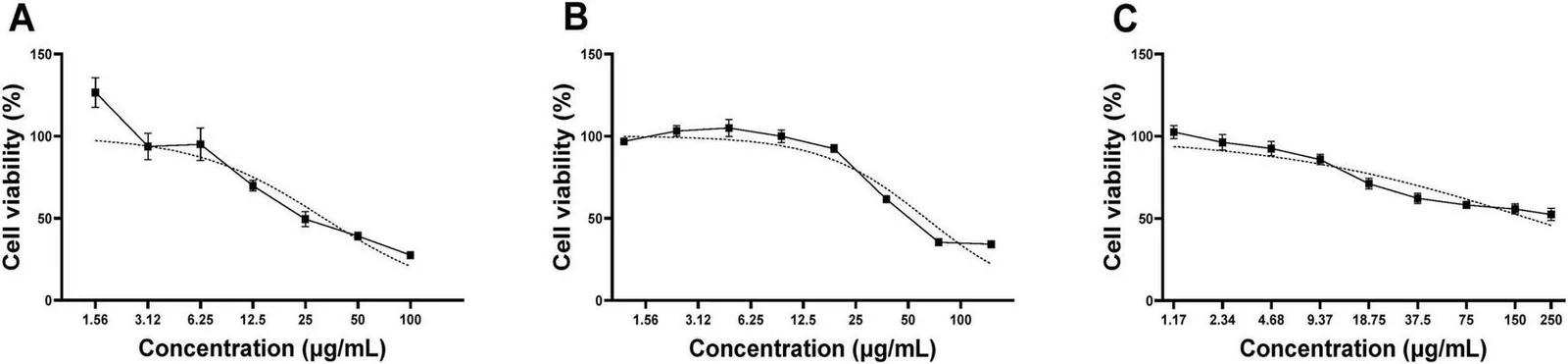
Dose-response curves of the ethyl acetate extract of the culture broth of strain S.100 (S.100 EtAc) against human cancer cells and normal cells after 48 h of treatment at a range of concentrations. **(A)** Cytotoxicity of S.100 EtAc on HeLa. **(B)** Cytotoxicity of S.100 EtAc on HepG2. **(C)** Cytotoxicity of S.100 EtAc on HEK 293T. Data represent mean ± SEM, *n* = 3 biological replicates (*p* < 0.05).

#### 3.3.2 Anti-inflammatory activities of S.100

S.100 EtAc displayed a remarkable anti-inflammatory activity at the concentration of 120 μg/mL. It significantly reduced NO production by 26.86% relative to the LPS-stimulated control (*p* < 0.05) (Figure 5).

**FIGURE 5:**
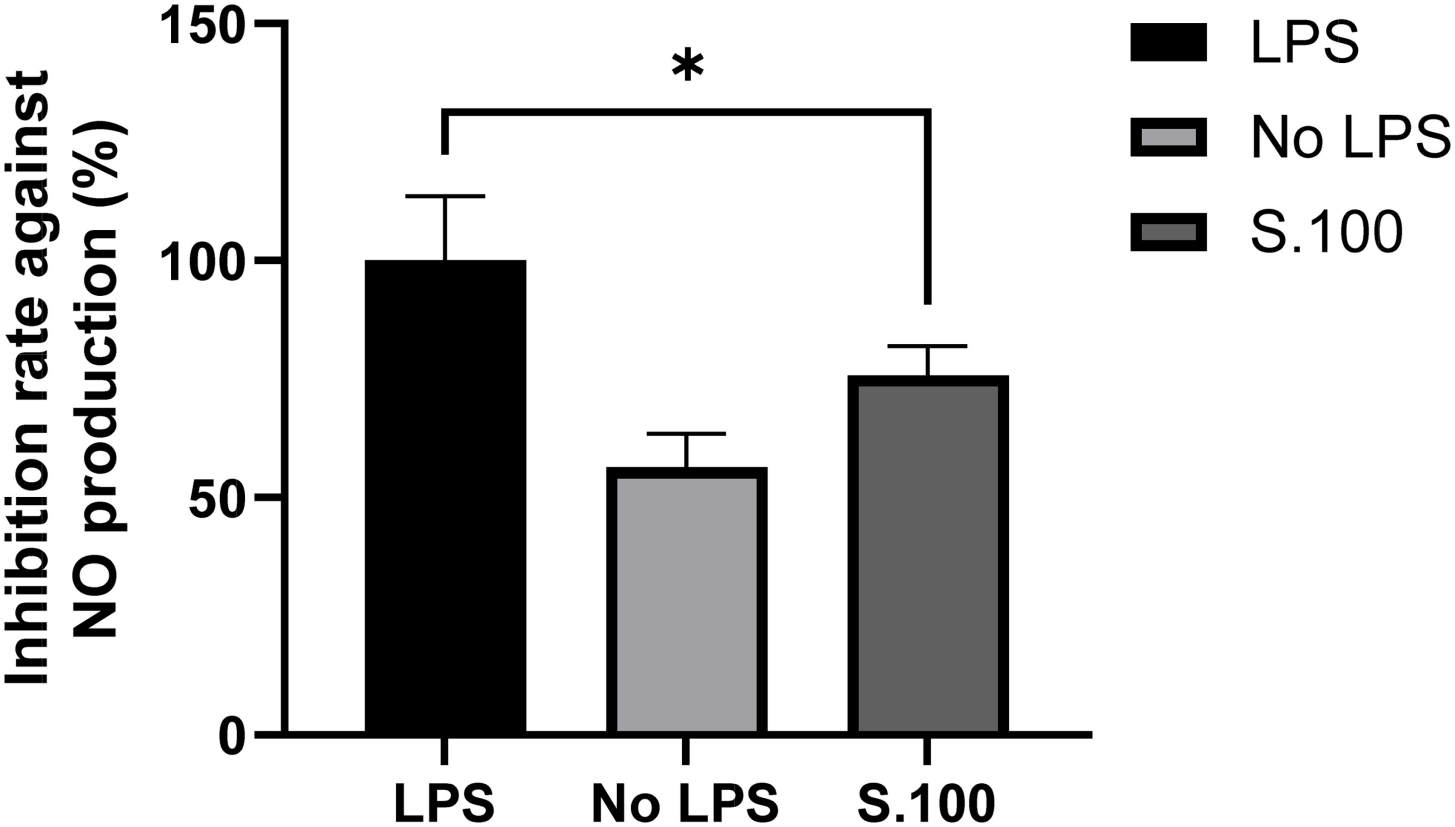
The anti-inflammatory activity of the ethyl acetate extract of the culture broth of strain S.100 (S.100 EtAc) at concentration of 120 μg/mL. Data represent mean ± SEM, *n* = 3 biological replicates (*p* < 0.05). The asterisk (*) indicates a statistically significant difference between the LPS-stimulated control and the LPS-stimulated cells treated with S.100 EtAc (120 μg/mL) (*p* < 0.05; one-way ANOVA).

#### 3.3.3 Zebrafish toxicological test

Treatment of S.100 EtAc with zebrafish embryos demonstrated concentration- and time-dependent toxicity, which was reflected in both lethality and developmental toxicity (Figure 6). At low concentrations of S.100 EtAc, i.e., in the range of 100–300 mg/L, survival rates of zebrafish embryos were high, and hatching rates and malformation were generally unaffected. In contrast, at the concentrations in the range of 400–500 mg/L, survival rates of zebrafish embryos decreased sharply, accompanied by a rapid increase in deformities and a decline in hatching rates at later stages (Figures 6A–D). The LC_50_ values decreased gradually from 920.5 mg/L at 24 h to 507.4 mg/L at 72 h and further to 421.6 mg/L at 96 h. This indicates that developmental toxicity emerged close to the lethal concentration.

**FIGURE 6:**
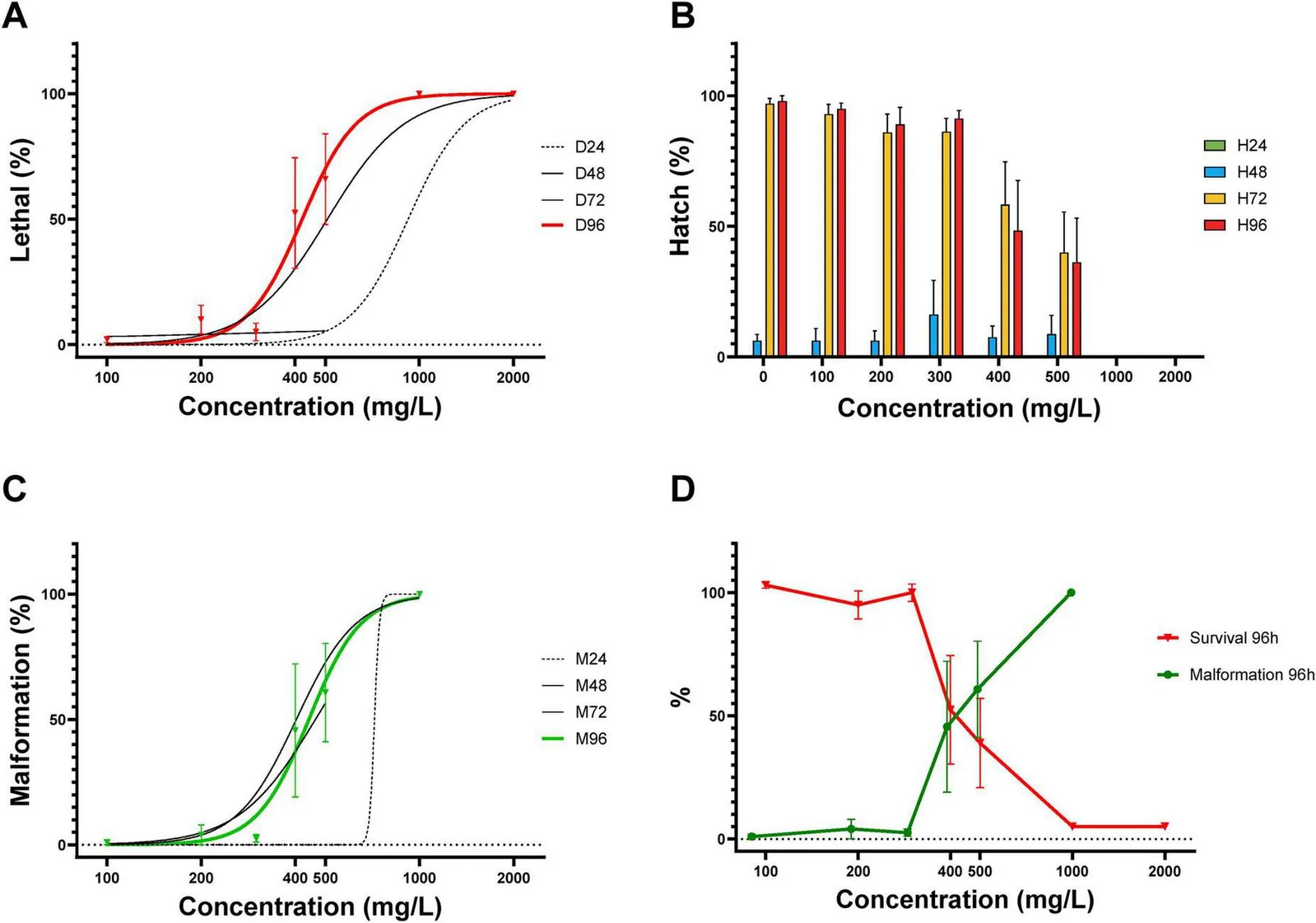
Concentration- and time-dependent toxicity of the ethyl acetate extract of the culture broth of strain S.100 (S.100 EtAc) on the zebrafish model. **(A)** The lethal toxicity curves of S.100 EtAc at 24, 48, 72, and 96 h (D24, D48, D72, and D96, respectively). The S.100 EtAc concentrations tested were 100, 200, 400, 500, 1000 and 2000 mg/L. **(B)** The hatch rate (% hatch) of zebrafish embryos after 24, 48, 72, and 96 h of exposure to varied concentrations of S.100 EtAc. **(C)** The malformation development curve (% malformation) at 24, 48, 72, and 96 h (M24, M48, M72, M96) in relation to varied S.100 EtAc concentrations. **(D)** The concentration-response relationship of survival rate (Survival, 96 h) and malformation rate (Malformation, 96 h) after S.100 EtAc exposure. Data represent mean ± SEM, *n* = 3 biological replicates (*p* < 0.05).

In addition, morphological observations of zebrafish embryos at higher concentrations, particularly in the later stages (72–96 hpf), exhibited more pronounced yolk sac edema and pericardial edema compared to controls and lower concentrations (Figure 7). Thus, S.100 EtAc caused cumulative toxicity over time, and the typical developmental damage is yolk edema and cardiac edema with inhibition of hatching at the concentration of 400–500 mg/L.

**FIGURE 7:**
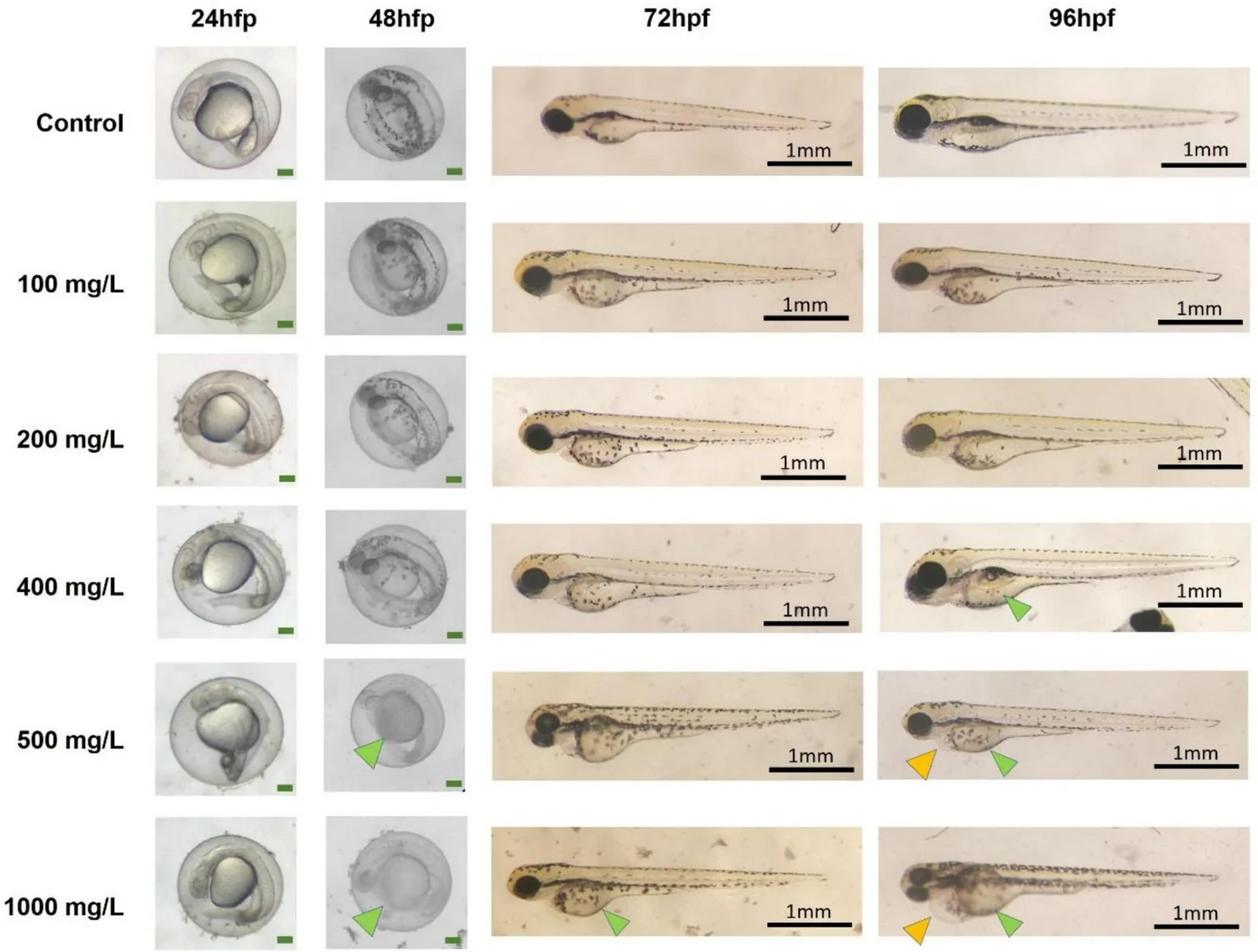
Morphological analysis of zebrafish embryos and larvae after certain periods of exposure to S.100 EtAc at varied concentrations: 100, 200, 400, 500, and 1,000 mg/L. The extract was conducted on zebrafish embryos at four endpoints: 24, 48, 72, and 96 h post-fertilization (hpf). Representative photos of the embryos were taken using a stereomicroscope. Green scale bar = 0.1 mm; black scale bar = 1 mm; yellow arrows indicate cardiac edema, green arrows indicate darkened yolk.

The results indicated that S.100 EtAc was well-tolerated by the embryos at concentrations below 200 mg/L in terms of morphological integrity, malformation incidence, hatching rate, and survival rate, consistent with the developmental toxicity thresholds recommended by the International Council for Harmonization ICH S5(R3) Guideline (Song et al., 2021).

### 3.4 Chemical characterization associated with the potential biopharmaceutical activities

As the results above show, S.100 EtAc has significant biopharmaceutical properties, including antiproliferative and anti-inflammatory activities. S.100 EtAc was therefore fractionated for analyzing the chemical compounds in this extract. The extract was separated into five fractions (designated as fractions 1–5) by utilizing methanol, n-hexane, acetone, and dichloromethane as solvents, with variations in their proportions and polarities. Upon evaluating the activities of the five fractions, we determined that fraction 4 demonstrated significant potential activities (Table 4 and Supplementary Figures 3–5). Therefore, we chose fraction 4 for LC-HRMS analysis to determine the chemicals present in it and to explore their structures.

**TABLE 4:** The biopharmaceutical activities of five fractions of S.100 EtAc extract.

Fraction	Antiproliferative activities		Anti-inflammatory activities	
	Against HeLa	Against HepG2	Cytotoxicity on RAW264.7	NO Griess Reagent
Fraction 1	−	−	−	−
Fraction 2	−	−	−	+
Fraction 3	−	−	−	+
Fraction 4	+	+	+	+
Fraction 5	+	−	−	+

“−” indicates weak activity, “+” indicates strong activity.

By analyzing the LC-HRMS data we putatively identified 16 metabolites in fraction 4 of S.100 EtAc extract, with their formulas and structures (Table 5). These chemical compounds belong to diverse groups including alkaloids (quinoline-4-carboxylic acid, norharmane, harmane), amino acids (DL-phenylalanine, L-(-)-tyrosine), vitamins [pantothenate (Vitamin B5), pantothenic acid], phenolic acids (2,3-dihydroxybenzoic acid, DL-4-hydroxyphenyllactic acid, gallic acid), fatty acids (16-hydroxyhexadecanoic acid), nucleosides (N6-methyladenosine), lipopeptides (surfactin C, surfactin B), and amino acid derivatives (N-acetyl-L-tyrosine, succinyl-isoleucine) (Table 5).

**TABLE 5:** The compounds found in fraction 4 of S.100 and their predicted chemical formula and structures.

No	t_R_ (min)	Compound name	Ion adduct	Observed mass (m/z)	Molecular formula (error in ppm)	Top 5 fragment ion (m/z)	Chemical structure
1	2.84	Quinoline-4-carboxylic acid	[M+H]+	174.055	C_10_H_7_NO_2_ (0.1)	174.0551, 130.065, 146.0602, 103.0547, 128.0497	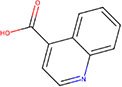
2	3.21	N6-methyladenosine	[M+H]+	282.12	C_11_H_15_N_5_O_4_ (1.3)	150.0776, 94.04044, 123.0667, 108.0435, 136.0621	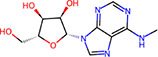
3	4.48	Pantothenate	[M−H]−	218.103	C_9_H_17_NO_5_ (0.1)	71.049, 88.039, 99.044, 146.081, 91.78	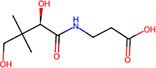
4	4.69	DL-phenylalanine	[M+H]+	166.0863	C_9_H_11_NO_2_ (2.3)	120.081, 103.055, 91.05486, 53.03951, 93.07049	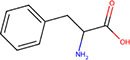
5	4.75	Pantothenic acid	[M+H]+	220.118	C_9_H_17_NO_5_ (0.6)	90.05558, 70.02957, 67.05506, 124.076, 116.0346	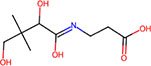
6	6.64	2,3-dihydroxybenzoic acid	[M−H]−	153.0183	C_7_H_6_O_4_ (3.9)	153.0184, 109.0280, 108.0204, 91.01762, 81.03321	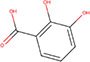
7	6.97	N-acetyl-L-tyrosine	[M−H]−	222.0768	C_11_H_13_NO_4_ (0.9)	58.029, 119.049, 107.049, 180.066, 93.033	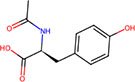
8	6.99	DL-4-hydroxyphenyllactic_acid	[M−H]−	181.05	C_9_H_10_O_4_ (6.7)	134.0362, 135.0441, 72.99181, 119.049, 163.0392	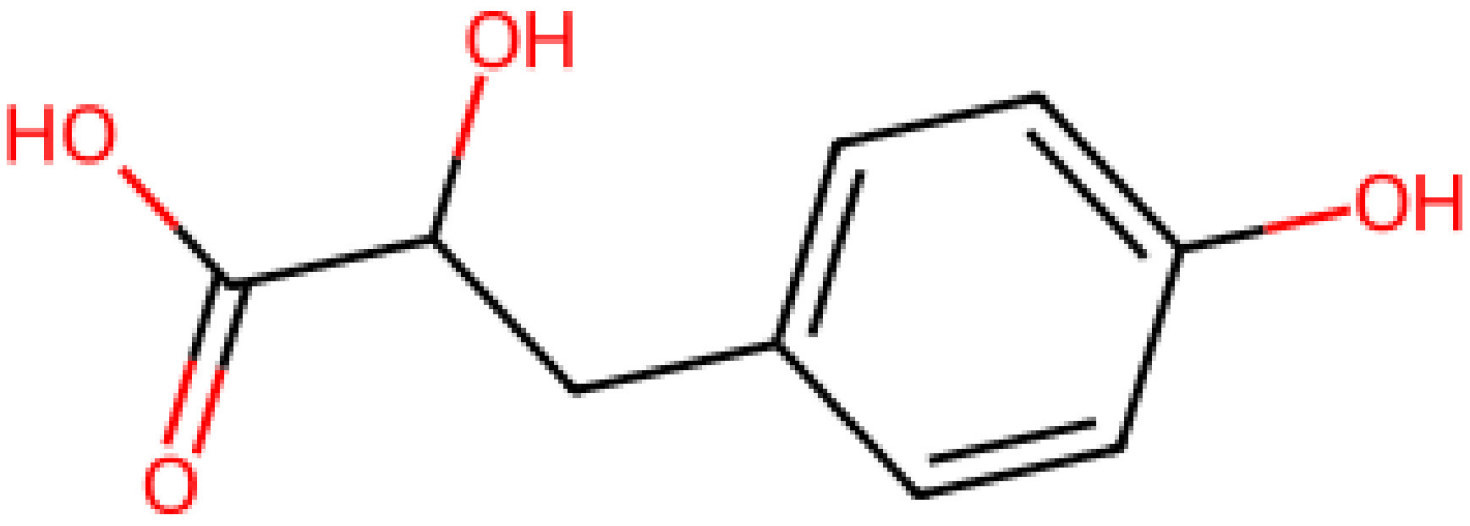
9	8.37	Norharmane	[M+H]+	169.0761	C_11_H_8_N_2_ (0.1)	169.0762, 115.0546, 168.0684, 142.0563, 56.96569	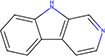
10	8.42	Gallic acid	[M−H]−	169.013	C_7_H_6_O_5_ (4.8)	69.033, 95.013, 124.015, 125.023, 97.028	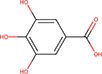
11	8.64	L-(-)-tyrosine	[M+H]+	182.081	C_9_H_11_NO_3_ (1.5)	91.05483, 136.0758, 123.0444, 95.0497, 119.0495	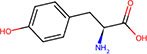
12	8.67	Harmane	[M+H]+	183.092	C_12_H_10_N_2_ (1.4)	183.0919, 115.0547, 168.0683, 70.20954, 142.0654	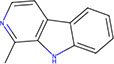
13	8.8	Succinyl-isoleucine	[M−H]−	230.103	C_10_H_17_NO_5_ (0)	130.086, 98.023, 80.964, 111.031, 168.1022	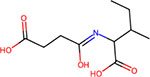
14	13.09	16-hydroxyhexadecanoic acid	[M+H−H_2_ O]+	255.2317	C_16_H_32_O_3_ (0.6)	55.05515, 69.07071, 57.0708, 83.08624, 81.07058	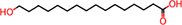
15	23.49	Surfactin C	[M+H]+	1036.6905	C_53_H_93_N_7_O_13_ (0.2)	86.09712, 84.04502, 72.08147, 91.77657, 199.1816	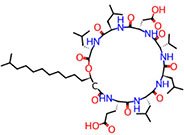
16	21.85	Surfactin B	[M+H]+	1022.6748	C_52_H_91_N_7_O_13_ (3.8)	86.09711, 84.04508, 72.0815, 91.77397, 201.1236	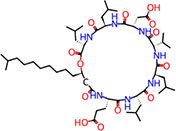

Among these, surfactin C and surfactin B are compounds secreted by *Bacillus* strains into the culture medium and have been confirmed by several previous studies (Ito et al., 2025). The other compounds might result from either the secretion of *B. stercoris* into the bacterial culture medium during its growth or the degradation of the bacterial cells.

## 4 Discussion

To the best of our knowledge, the present study reports, for the first time, the association of *B. stercoris* with the red alga *G. tenuistipitata* collected from Vietnamese coastal waters. This claim is based on our systematic search across multiple databases including PubMed, Web of Science, Scopus, and Google Scholar, with no date restriction, i.e., the last search was in August 2026. Previous surveys of seaweed-associated bacterial communities have documented that bacteria associated with *Gracilaria* spp. are predominantly affiliated with the genera *Pseudoalteromonas, Vibrio, Bacillus*, and *Flavobacterium* (Egan et al., 2013; Hollants et al., 2013). While the genus *Bacillus* is thus well-established as a component of the *Gracilaria* microbiome, the species *B. stercoris* has not previously been reported from any seaweed host, which makes the finding in this study particularly interesting. The discovery of *B. stercoris* in association with *G. tenuistipitata* enriches our understanding of algal-associated bacterial diversity in Vietnamese coastal waters. It may also imply certain symbiotic mechanisms that may partly account for the bioactivities of the algae. It has long been recognized that the pharmacological properties of algae do not derive solely from the algal tissues themselves but frequently result from complex interactions between them, the hosts, and their associated microbial communities (Egan et al., 2013; Singh and Reddy, 2016).

The discovery of *B. stercoris* from *G. tenuistipitata* is also interesting because in general, *B. stercoris* is often found in soil or environmental samples rich in organic compounds, including food waste treatment bioreactors (Yu et al., 2024) and agricultural soils (Ku et al., 2024; Pengproh et al., 2023). *B. stercoris* has been also found associated with plants due to various outstanding beneficial properties this bacterium offers to plants, such as promoting plant growth (Yu et al., 2024), degrading cellulose (Singh et al., 2024), and producing biocontrol substances to protect plants from diseases (Wang et al., 2021). Previous research on *B. stercoris* genome has revealed the presence of several important genes involved in the synthesis of enzymes capable of breaking down complex organic compounds, the synthesis of plant hormones, the breakdown of polysaccharides, and phosphate solubilization, which are processes favoring plant wellbeing (Wang et al., 2021). In this study, the isolation of *B. stercoris* from *G. tenuistipitata* indicates that this bacterial species possesses the ability to coexist symbiotically with the seaweed in the harsh conditions of the deep-sea environment and may benefit the seaweed in the same ways. Furthermore, members of the genus *Bacillus* have been repeatedly reported in the literature as functional symbionts of macroalgae; for instance, endophytic *Bacillus pumilus* and *Bacillus licheniformis* isolated from the red alga *Gracilaria dura* were shown to produce indole-3-acetic acid and to contribute to bud induction and growth of the algal host (Singh et al., 2011).

*G. tenuistipitata* is a red alga widely cultivated in tropical and subtropical regions, including Vietnam, and contributes substantially to global agar production (Titlyanov et al., 2012; Torres et al., 2019). Previous studies have clearly demonstrated the anti-inflammatory and anti-cancer potentials of extracts from this seaweed. Its extracts have been also shown to protect against oxidative stresses and inhibit cell proliferation in several cell lines, such as H1299 non-small-cell lung cancer cells (Yang et al., 2012), oral cancer Ca9-22 cells (Yeh et al., 2012). More recently, enzymatic hydrolysates derived from phycobiliproteins of the alga have demonstrated their strong antioxidant and anti-inflammatory activities (Khan et al., 2026). There has been no clear evidence whether these activities are attributed to the alga itself or may be related to their associated microorganisms but the results of recent studies support the latter. Thus, the discovery of *B. stercoris* S.100 from *G. tenuistipitata* suggests that this seaweed-associated bacterium may play a significant role in contributing to or enhancing the observed bioactivities of the alga. This hypothesis is in line with the anti-inflammatory and antiproliferative activities of *B. stercoris* S.100.

Although the data demonstrated a remarkable anti-inflammatory activity of *B. stercoris* S.100, it should be noted that nitric oxide (NO) inhibition was used in this study as a primary screening readout for anti-inflammatory activity, given its wide validation in LPS-stimulated macrophage models. However, NO inhibition alone does not fully capture the anti-inflammatory potential of strain S.100, as it does not reflect effects on other inflammatory mediators or pathways. The present findings should therefore be regarded as preliminary evidence of anti-inflammatory potential, warranting further validation through additional markers such as TNF-α, IL-6, IL-1β, iNOS, and COX-2 in future studies.

Among the bioactivities of *B. stercoris* S.100, antiproliferation is probably the most impressive. As stated above in the cytotoxicity test results, the IC_50_ values of S.100 EtAc extract were 31.87, 63.8, and 181.1 μg/mL for HeLa, HepG2, and HEK 293T cells, respectively. According to the cytotoxic activity classification criteria of Cos et al. (2006), extracts with IC_50_ values in the range of 20–100 μg/mL are considered moderately cytotoxic. Therefore, the extract can be considered to have moderate toxicity against both cancer cell lines, with HeLa demonstrating approximately twice the sensitivity compared to HepG2. The resulting SI values were 5.68 for HeLa and 2.84 for HepG2, both exceeding the threshold of SI ≥ 2 commonly adopted in natural product cytotoxicity screening as indicative of biologically meaningful selectivity (Monks et al., 1991). The SI of 5.68 against HeLa is particularly noteworthy and warrants further mechanistic investigation. Thus, the result indicates a higher selectivity for cancer cells over non-cancer cells; however, the translation of these *in vitro* findings to *in vivo* applications would require further investigation.

With such promising bioactivity potential, it is even more encouraging that *B. stercoris* S.100 can be considered safe for use. Noteworthily, *B. stercoris* belongs to the *B. subtilis sensu lato* group, which is classified as “Generally Recognized as Safe” by FDA (The U.S Food Drug Administration [FDA], 2018). Furthermore, its safety is supported by the above-stated results of our toxicity test on the zebrafish embryo model. The zebrafish embryo model has been widely adopted for toxicological evaluation of natural product extracts, offering important ethical and practical advantages over mammalian *in vivo* models (OECD, 2013). Thus *B. stercoris* S.100 satisfies the safety rationale for further preclinical investigation and its biopharmaceutical potential appears even more promising.

The observed bioactivities of *B. stercoris* S.100 are hypothesized to be associated with the secondary metabolites it secretes, although the present data do not establish a direct causal link between individual compounds and the observed activities. Indeed, seaweed-associated bacteria of the genus *Bacillus* are well-established producers of a diverse array of bioactive secondary metabolites, including lipopeptides, polyketides, and volatile organic compounds (Goecke et al., 2010). In particular, *B. stercoris* is phylogenetically affiliated with the *B. subtilis* species complex, members of which are well-characterized producers of cyclic lipopeptides including surfactin, iturin, and fengycin, as well as polyketides, the antimicrobial peptide bacilysin, and the siderophore bacillibactin (Raaijmakers et al., 2010; Stein, 2005). In our study, the LC-HRMS identification of surfactin B and surfactin C in fraction 4 of the S.100 EtAc extract provides evidence suggesting that lipopeptide biosynthesis occurs in this strain. In the context of the host–microbe relationship, *B. stercoris* may additionally participate in modifying or upregulating polysaccharide, polyphenol, and exopolysaccharide (EPS) biosynthesis in the algal host, thereby indirectly enhancing the overall bioactivity of *G. tenuistipitata* (Egan et al., 2013).

A total of 16 compounds from fraction 4 of the S.100 EtAc extract were putatively identified via LC-HRMS (Table 5). Several of those compounds, including quinoline-4-carboxylic acid, N6-methyladenosine, norharmane, gallic acid, DL-4-hydroxyphenyllactic acid, succinyl-isoleucine, and 16-hydroxyhexadecanoic acid, have not previously been reported from *Bacillus* spp. associated with *G. tenuistipitata*, and their occurrence in *B. stercoris* thus warrants further characterisation. Norharmane is a β-carboline alkaloid reported to possess neuroactive, receptor-binding, and anticancer properties in multiple experimental systems (Farzin and Mansouri, 2006; Foley et al., 2019; Zheng et al., 2006). Gallic acid is a well-characterized polyphenolic antioxidant with documented anti-inflammatory activity mediated through inhibition of cytokine production, COX-2 expression, and NF-κB signaling, as well as hepatoprotective activity demonstrated in animal models (Kahkeshani et al., 2019; Verma et al., 2013). Particularly noteworthy are surfactin B and surfactin C, which represent the most direct experimental evidence linking the bioactivities of S.100 to the lipopeptide biosynthetic capacity of *B. stercoris*. These lipoheptapeptides were first isolated and characterized from culture media of *B. subtilis* (Arima et al., 1968) and have since been confirmed as products of multiple *Bacillus* strains by HPLC and LC-MS analyses (Raaijmakers et al., 2010; Stein, 2005). The documented biological activities of surfactins encompass antibacterial effects (Ongena and Jacques, 2008), antifungal activity (Ongena and Jacques, 2008), and anti-inflammatory activity mediated through inhibition of phospholipase A2 (PLA2), suppression of NF-κB signaling, and reduction of pro-inflammatory cytokine production (Cho et al., 2006; Madhi and Kim, 2026; Raaijmakers et al., 2010). With respect to anticancer properties, surfactins have been shown to inhibit cancer cell proliferation, induce mitochondria-dependent apoptosis through ROS accumulation and mitochondrial membrane potential collapse, suppress metastatic migration and invasion, and downregulate matrix metalloproteinases including MMP-9 across multiple cancer cell line models (Kim et al., 2007; Wang et al., 2013). These established activities of surfactins raise the hypothesis that surfactin B and C may partly contribute to the anti-inflammatory and cytotoxic activities observed against HeLa and HepG2 cells in the present study; however, since fraction 4 contains multiple co-occurring metabolites, further studies using purified/isolated compounds are needed to establish a direct causal relationship between specific compounds and the observed bioactivities.

## 5 Conclusion

In this study, we report for the first time the presence of *B. stercoris* living symbiotically with the red alga *G. tenuistipitata*. The isolated *B. stercoris* strain S.100 displayed noteworthy anti-inflammatory and antiproliferative activities through the respective assays with its ethylacetate extract. Particularly, the extract exerts a higher selective cytotoxicity toward HeLa and HepG2 cancer cells over non-cancer cells. The bioactivities of S.100 were highly attributed to the metabolites it produces, which include 16 compounds in the ethylacetate extract, identified by using LC-HRMS, as many of them are known to possess significant anti-inflammatory and antiproliferative activities. These findings suggest that *B. stercoris* may importantly contribute to the bioactivities of the host *G. tenuistipitata* and has promising biopharmaceutical potential for future applications.

## Data Availability

The datasets presented in this study can be found in online repositories. The names of the repository/repositories and accession number(s) can be found in the article/Supplementary material.
