# Supplementary material for: A novel bacterial symbiont in the seaweed Gracilaria tenuistipitata and its promising anti-inflammatory and antiproliferative potential in relation to special secreted metabolites
Source: Front Microbiol. 2026 Sep 29;17:1935663. doi: 10.3389/fmicb.2026.1935663 (PMC13657998; doi:10.3389/fmicb.2026.1935663)
Supplement: Supplementary file 1 [file Data_Sheet_1.docx]

Supplementary Material

**A novel bacterial symbiont in the seaweed *Gracilaria tenuistipitata* and its promising anti-inflammatory and antiproliferative potential in relation to special secreted metabolites**

By Anh Hien Le et al.

**Additional information about methods**

*The method for isolation of seaweed-associated microorganisms used in our previous studies*

Seaweed samples were washed three times with sterile water to remove free-living and loosely attached bacteria. For isolation of endophytic microorganisms, seaweed tissues were surface-sterilized sequentially with 96% ethanol for 3 min, 3% H₂O₂ for 3 min, and 1% NaClO for 3 min, followed by thorough rinsing with sterile water. As a sterility control, an aliquot of the final rinse water was plated on the same isolation media; no microbial growth was observed on these control plates, confirming the effectiveness of the surface-sterilization procedure and supporting the internal origin of the isolates obtained from the sterilized tissue. The surface-sterilized tissues were then aseptically homogenized in sterile water, and microorganisms were isolated by serial dilution in 0.9% NaCl solution, plated in triplicate on eight agar media differing in nutrient and salinity conditions (LB, LB 3% NaCl, YS, YS 3% NaCl, PDA, PDA 3% NaCl, Marine Agar, and SCA), and incubated at 30 °C for at least 7 days.

*Raw LC-HRMS data processing and analysis*

Raw mass spectrometry data were initially captured in vendor-specific RAW format and afterwards transformed into mzML format with MSConvert. Data pre-processing was conducted utilizing MZmine version 4.8.0. The first 0.00–1.00 minutes of each chromatogram were discarded to remove solvent-related and early-eluting background signals. Mass detection was performed in centroid mode with noise thresholds established at 5.0 × 10³ for MS¹ scans and 2.5 × 10¹ for MS² scans, resulting in a preliminary mass list. Chromatograms were generated from the identified features with the ADAP chromatogram builder, requiring a minimum of four consecutive scans, an intensity threshold of 10,000 for consecutive scans, and a minimum peak height of 50,000. The m/z tolerance for chromatogram construction was established at 10 ppm. Isotopic characteristics were categorized and refined utilizing the ¹³C isotope filter in MZmine, with a m/z tolerance of 0.01 Da (3 ppm) and a retention time tolerance of 0.1 min. Only features with charge statuses of up to 2 were taken into consideration. The most intense isotope was maintained as the typical characteristic, but properties related to MS² spectra were retained. Feature alignment across analytical runs was conducted utilizing the Join Aligner in MZmine, applying a m/z tolerance of 0.01 Da (5 ppm) and a retention time tolerance of 0.1 min. Subsequent to feature alignment, features were subjected to a sample-based row filter, preserving only those identified in a minimum of two aligned samples, indicative of characteristics consistently found throughout duplicate LC–HRMS studies. Potential duplicate features were subsequently removed using the Duplicate Peak Filter, applying strict m/z (0.005 Da, 1.5 ppm) and retention time (0.07 min) tolerances. Spectral library searching was conducted in MZmine to putatively annotate compounds using MS² spectra derived from the aligned feature list and compared against reference MS/MS spectral libraries. Prior to library matching, MS² spectra were merged, and spectral comparisons were performed against imported public reference MS/MS libraries, including GNPS spectral libraries, ENAMDISK, MCE Bioactive, NIH Natural Products, and MassBank libraries, in both positive and negative electrospray ionization modes. The precursor ion m/z tolerance was set to 0.01 Da (10 ppm), and the fragment ion m/z tolerance was set to 0.01 Da (10 ppm). Library annotations were accepted only when at least six fragment ions were matched and a weighted cosine similarity score of ≥ 0.70 was achieved. According to the Metabolomics Standards Initiative (MSI), all annotated compounds were classified as putatively annotated metabolites (MSI Level 2) [65], as identifications were based on accurate precursor mass, MS/MS spectral similarity to reference libraries, and consistent fragmentation patterns, but were not confirmed using authentic reference standards analyzed under identical experimental conditions. Subsequent removal of any duplicate features was executed using the Duplicate Peak Filter, with stringent limits for m/z (0.005 Da, 1.5 ppm) and retention period (0.07 min). Spectral library searching was performed in MZmine to tentatively annotate compounds utilizing MS² spectra obtained from the aligned feature set, which were compared against reference MS/MS spectral libraries. Before library matching, MS² spectra were consolidated, and spectral comparisons were conducted against imported public reference MS/MS libraries, including GNPS spectral libraries, ENAMDISK, MCE Bioactive, NIH Natural Products, and MassBank libraries, in both positive and negative electrospray ionization modes. The m/z tolerance for the precursor ion was established at 0.01 Da (10 ppm), and the m/z tolerance for the fragment ion was also set at 0.01 Da (10 ppm). Library comments were permitted only when a minimum of six fragment ions were matched and a weighted cosine similarity score of ≥ 0.70 was attained. The Metabolomics Standards Initiative (MSI) classified all annotated compounds as putatively annotated metabolites (MSI Level 2) [17], as identifications relied on precise precursor mass, MS/MS spectral similarity to reference libraries, and consistent fragmentation patterns, but were not validated with authentic reference standards analyzed under identical experimental conditions.

**Supplemental data**

**Supplementary Figure 1. Cytotoxicity Screening of Strain S.100 Extracts (150 µg/mL) on HeLa and HepG2 Cell Lines.**

**Supplementary Figure 2. Dose-response curves of** **doxorubicin against human cancer cells after 48 hours of treatment at various concentrations: 20, 2, 1, 0.2, 0.02, 0.002 and 0.0002 µg/mL.** *Notes*: (A) Cytotoxicity of Doxorubicin on HeLa, (B) Cytotoxicity of Doxorubicin on HepG2. Data represent mean ± SEM, n = 3 biological replicates. (P < 0.05).

**Supplementary Figure 3. Dose-response curves of five fractions from the S.100 extract against HeLa cells at various concentrations (1, 10, 25, 50, and 100 µg/mL).** *Notes*: (A–E) Cytotoxicity of Fractions 1–5, respectively. Data represent mean ± SEM, n = 3 biological replicates. (P < 0.05)

**Supplementary Figure 4. Dose-response curves of five fractions from the S.100 extract against HepG2 cells at various concentrations (1, 10, 25, 50, and 100 µg/mL).** *Notes*: (A–E) Cytotoxicity of Fractions 1–5, respectively. Data represent mean ± SEM, n = 3 biological replicates. (P < 0.05)

**Supplementary Figure 5. Effects of five fractions from the S.100 extract on RAW 264.7 macrophages at various concentrations (1, 25, 50, and 100 µg/mL).** *Notes*: (A–E) Cell cytotoxicity curves of Fractions 1–5. (F–K) Anti-inflammatory activity (inhibition rate against NO production) of Fractions 1–5, respectively. Data represent mean ± SEM, n = 3 biological replicates. (P < 0.05)

A and F – Fraction 1

B and G – Fraction 2

C and H – Fraction 3

D and I – Fraction 4

E and K – Fraction 5

**Cell viability data**

The complete raw data are presented below, and viability (%) values were calculated using the following formula (already stated in the Materials and Methods section):

$Cell Viability (\%)=\left( \frac{OD\_sample}{OD control} \right)x100$

During data acquisition, the absorbance of extract-only blanks was measured in parallel and subtracted from the corresponding treatment wells at the time of reading; therefore, the reported absorbance/viability values are already blank-corrected, and no additional blank subtraction was performed in subsequent calculations.

**Supplementary Table 1. Raw OD570 nm values used to generate the dose-response curves of the EtAc extract of the culture broth of strain S.100 in HepG2 cells.** Note: R1, R2, R3: the 3 replicates

| Concentration (μg/mL) | R1 | | | R2 | | | R3 | | |
| --- | --- | --- | --- | --- | --- | --- | --- | --- | --- |
| Untreated control | 0.95 | 0.938 | 0.959 | 0.89 | 1.019 | 0.931 | 0.664 | 0.626 | 0.754 |
| Vehicle control | 0.952 | 0.867 | 0.881 | 0.934 | 1.014 | 1.323 | 0.6 | 0.623 | 0.709 |
| 1.171875 | 0.88 | 0.892 | 0.879 | 1.012 | 1.007 | 1.058 | 0.698 | 0.628 | 0.647 |
| 2.34375 | 0.799 | 0.847 | 1.064 | 1.209 | 1.027 | 1.032 | 0.728 | 0.758 | 0.632 |
| 4.6875 | 0.807 | 1.05 | 0.316 | 1.183 | 1.237 | 0.865 | 0.793 | 0.759 | 0.648 |
| 9.375 | 0.821 | 0.896 | 0.966 | 1 | 1.065 | 0.995 | 0.728 | 0.799 | 0.652 |
| 18.75 | 0.654 | 0.738 | 0.724 | 0.972 | 0.959 | 0.978 | 0.581 | 0.692 | 0.649 |
| 37.5 | 0.619 | 0.638 | 0.529 | 0.687 | 0.742 | 0.639 | 0.382 | 0.363 | 0.378 |
| 75 | 0.329 | 0.366 | 0.354 | 0.345 | 0.351 | 0.351 | 0.282 | 0.271 | 0.262 |
| 150 | 0.353 | 0.348 | 0.345 | 0.349 | 0.345 | 0.343 | 0.262 | 0.252 | 0.242 |

**Supplementary Table 2. Normalized cell viability of HepG2 cells following treatment with the EtAc extract from the culture broth of strain S.100.** Note: R1, R2, R3: the 3 replicates

| Concentration (μg/mL) | R1 | | | R2 | | | R3 | | |
| --- | --- | --- | --- | --- | --- | --- | --- | --- | --- |
| 1.171875 | 92.82 | 92.36 | 97.03 | 92.82 | 92.36 | 97.03 | 108.39 | 97.52 | 100.47 |
| 2.34375 | 110.88 | 94.19 | 94.65 | 110.88 | 94.19 | 94.65 | 113.04 | 117.70 | 98.14 |
| 4.6875 | 108.50 | 113.45 | 79.33 | 108.50 | 113.45 | 79.33 | 123.14 | 117.86 | 100.62 |
| 9.375 | 91.72 | 97.68 | 91.26 | 91.72 | 97.68 | 91.26 | 113.04 | 124.07 | 101.24 |
| 18.75 | 89.15 | 87.95 | 89.70 | 89.15 | 87.95 | 89.70 | 90.22 | 107.45 | 100.78 |
| 37.5 | 63.01 | 68.05 | 58.61 | 63.01 | 68.05 | 58.61 | 59.32 | 56.37 | 58.70 |
| 75 | 31.64 | 32.19 | 32.19 | 31.64 | 32.19 | 32.19 | 43.79 | 42.08 | 40.68 |
| 150 | 32.01 | 31.64 | 31.46 | 32.01 | 31.64 | 31.46 | 40.68 | 39.13 | 37.58 |

**Supplementary Table 3. Raw OD570 nm values used to generate the dose-response curves of the EtAc extract of the culture broth of strain S.100 in Hela cells.** Note: R1, R2, R3: the 3 replicates

| Concentration (μg/mL) | R1 | | | R2 | | | R3 | | |
| --- | --- | --- | --- | --- | --- | --- | --- | --- | --- |
| Untreated control | 0.919 | 0.948 | 0.823 | 1.131 | 1.026 | 0.925 | 0.864 | 0.707 | 0.842 |
| Vehicle control | 0.905 | 0.741 | 0.705 | 1.036 | 0.988 | 0.958 | 0.841 | 0.819 | 0.749 |
| 1.5625 | 1.139 | 1.045 | 1.22 | 1.131 | 1.026 | 0.925 | 0.705 | 0.95 | 0.828 |
| 3.125 | 0.765 | 0.787 | 1.14 | 1.036 | 0.988 | 0.958 | 0.611 | 0.676 | 0.692 |
| 6.25 | 0.825 | 0.848 | 1.213 | 1.131 | 1.026 | 0.925 | 0.597 | 0.596 | 0.59 |
| 12.5 | 0.483 | 0.453 | 0.5 | 1.036 | 0.988 | 0.958 | 0.623 | 0.603 | 0.565 |
| 25 | 0.239 | 0.235 | 0.272 | 1.131 | 1.026 | 0.925 | 0.494 | 0.489 | 0.473 |
| 50 | 0.237 | 0.279 | 0.232 | 1.036 | 0.988 | 0.958 | 0.367 | 0.372 | 0.387 |
| 100 | 0.25 | 0.259 | 0.223 | 1.131 | 1.026 | 0.925 | 0.206 | 0.227 | 0.299 |

**Supplementary Table 4. Normalized cell viability of Hela cells following treatment with the EtAc extract from the culture broth of strain S.100.** Note: R1, R2, R3: the 3 replicates

| Concentration (μg/mL) | R1 | | | R2 | | | R3 | | |
| --- | --- | --- | --- | --- | --- | --- | --- | --- | --- |
| 1.5625 | 145.34 | 133.35 | 155.68 | 103.32 | 95.27 | 98.59 | 87.79 | 118.31 | 103.11 |
| 3.125 | 97.62 | 100.43 | 145.47 | 63.48 | 73.44 | 85.51 | 76.09 | 84.18 | 86.18 |
| 6.25 | 105.27 | 108.21 | 154.79 | 76.96 | 68.61 | 92.76 | 74.35 | 74.22 | 73.47 |
| 12.5 | 61.63 | 57.81 | 63.80 | 64.99 | 66.50 | 89.44 | 77.58 | 75.09 | 70.36 |
| 25 | 30.50 | 29.99 | 34.71 | 49.70 | 53.22 | 64.99 | 61.52 | 60.90 | 58.90 |
| 50 | 30.24 | 35.60 | 29.60 | 36.72 | 37.42 | 41.65 | 45.70 | 46.33 | 48.19 |
| 100 | 31.90 | 33.05 | 28.46 | 24.35 | 20.42 | 18.01 | 25.65 | 28.27 | 37.24 |

**Supplementary Table 5. Raw OD570 nm values used to generate the dose-response curves of the EtAc extract of the culture broth of strain S.100 in HEK cells.** Note: R1, R2, R3: the 3 replicates

| Concentration (μg/mL) | R1 | | | R2 | | | R3 | | |
| --- | --- | --- | --- | --- | --- | --- | --- | --- | --- |
| Untreated control | 0.515 | 0.643 | 0.485 | 0.997 | 0.892 | 0.971 | 1.028 | 1.06 | 0.929 |
| Vehicle control | 0.501 | 0.642 | 0.531 | 1.042 | 0.843 | 0.932 | 1.069 | 1.069 | 1.079 |
| 2.34375 | 0.472 | 0.498 | 0.555 | 0.978 | 1.104 | 1.066 | 0.829 | 0.886 | 1.045 |
| 4.6875 | 0.475 | 0.449 | 0.636 | 1.009 | 0.807 | 0.964 | 0.9 | 0.831 | 1.027 |
| 9.375 | 0.48 | 0.481 | 0.558 | 0.731 | 0.791 | 0.914 | 0.805 | 0.788 | 0.985 |
| 18.75 | 0.403 | 0.369 | 0.426 | 0.617 | 0.636 | 0.865 | 0.669 | 0.646 | 0.823 |
| 37.5 | 0.367 | 0.348 | 0.397 | 0.554 | 0.552 | 0.723 | 0.496 | 0.566 | 0.705 |
| 75 | 0.312 | 0.338 | 0.367 | 0.487 | 0.549 | 0.541 | 0.38 | 0.678 | 0.559 |
| 150 | 0.258 | 0.33 | 0.391 | 0.482 | 0.513 | 0.658 | 0.505 | 0.513 | 0.586 |
| 250 | - | - | - | 0.499 | 0.477 | 0.616 | 0.446 | 0.467 | 0.637 |

**Supplementary Table 6. Normalized cell viability of HEK cells following treatment with the EtAc extract from the culture broth of strain S.100.**

| Concentration (μg/mL) | R1 | | | R2 | | | R3 | | |
| --- | --- | --- | --- | --- | --- | --- | --- | --- | --- |
| 2.34375 | 84.59 | 89.25 | 99.46 | 104.15 | 117.57 | 113.53 | 77.31 | 82.62 | 97.45 |
| 4.6875 | 85.13 | 80.47 | 113.98 | 107.45 | 85.94 | 102.66 | 83.93 | 77.49 | 95.77 |
| 9.375 | 86.02 | 86.20 | 100.00 | 77.85 | 84.24 | 97.34 | 75.07 | 73.48 | 91.86 |
| 18.75 | 72.22 | 66.13 | 76.34 | 65.71 | 67.73 | 92.12 | 62.39 | 60.24 | 76.75 |
| 37.5 | 65.77 | 62.37 | 71.15 | 59.00 | 58.79 | 77.00 | 46.25 | 52.78 | 65.74 |
| 75 | 55.91 | 60.57 | 65.77 | 51.86 | 58.47 | 57.61 | 35.44 | 63.23 | 52.13 |
| 150 | 46.24 | 59.14 | 70.07 | 51.33 | 54.63 | 70.07 | 47.09 | 47.84 | 54.65 |
| 250 | - | - | - | 53.14 | 50.80 | 65.60 | 41.59 | 43.55 | 59.40 |

**Supplementary Figure 6. Assessment of potential interference of S.100 EtAc extract with MTT absorbance.** *Notes*: “Only medium” = culture medium alone; “Blank” = medium + MTT reagent without extract. Data are presented as mean ± SD (n = 3). The MTT assays were performed without cells, only S.100 EtAc were added to the plate, The data showed that the OD values of all S.100 concentrations were comparable to the Blank control.
